# UK Preschool-aged children’s physical activity levels in childcare and at home: a cross-sectional exploration

**DOI:** 10.1186/s12966-015-0286-1

**Published:** 2015-09-26

**Authors:** Kathryn R. Hesketh, Simon J. Griffin, Esther M. F. van Sluijs

**Affiliations:** MRC Epidemiology Unit & Centre for Diet and Activity Research (CEDAR), University of Cambridge School of Clinical Medicine, Box 285 Institute of Metabolic Science, Cambridge Biomedical Campus, Cambridge, CB2 0QQ UK; Primary Care Unit, Cambridge Institute of Public Health, University of Cambridge School of Clinical Medicine, Cambridge, CB2 0SR UK; UCL Institute of Child Health, 30 Guilford Street, London, WC1N1EH UK

**Keywords:** Physical activity, Preschool children, Childcare, Home environment

## Abstract

**Background:**

Young children are thought to be inactive in childcare, but little is known about location-specific activity levels. This observational study sought to describe the in-care and out-of-care activity patterns of preschool-aged children and explore differences in physical activity level by childcare attendance.

**Methods:**

Three to four-year-old children were recruited from 30 preschool and nursery ‘settings’ in Cambridgeshire, UK. Average minutes per hour (min/h) spent sedentary (SED), in light physical activity (LPA) and in moderate-to-vigorous PA (MVPA) were measured by accelerometry for up to 7 days (mean: 6.7 ± 1.1). Weekly childcare attendance patterns were reported by parents. The within-child association between childcare attendance and outcomes was assessed using two- and three-level hierarchical regression; sex by care (in/out) interactions were considered.

**Results:**

Two hundred and two children (51 % female) had valid activity data for ≥2 days. Children, and particularly boys, were less sedentary and more active when in care compared to at home (SED: Boys: β (SE): −6.4 (0.5) min/h, Girls: −4.8 (0.5); LPA: Boys: 0.6 (0.4), Girls: 1.8 (0.4); MVPA: Boys: 5.7 (0.5); Girls: 3.0 (0.4)). Differences between in-care and at-home activity were largest in the (early) mornings and early evenings for boys; no compensation in at-home activity occurred later in the day. On days when children were in care part-time (1–5 h) or full-time (>5 h), they were significantly less sedentary and more active compared with non-care days.

**Conclusions:**

Young children, and particularly boys, accumulate more MVPA in care compared to at home. Future research should identify factors accounting for this difference and consider targeting non-care time in intervention efforts to increase higher-intensity activity and decrease sedentary time in preschoolers.

**Electronic supplementary material:**

The online version of this article (doi:10.1186/s12966-015-0286-1) contains supplementary material, which is available to authorized users.

## Introduction

Higher levels of physical activity in preschool-aged children have been shown to be associated with decreased adiposity, improved motor skill development, better psychosocial health, and favorable cardio-metabolic risk indicators [1]. Recently, countries worldwide (Canada [2]; Australia [3]; the UK [4]; and the USA [5]) have developed activity guidelines for children under 5 years old. Most recommend that children engage in at least 180 min of any activity daily (including light (LPA) and moderate to vigorous physical activity (MVPA)) [2–4]. Estimates of the proportion of children meeting these guidelines vary (e.g. 5 % in Australia [6]; 84 % in Canada [7]; 100 % in the UK [8]), at least in part due to differences in measurement and post-processing protocols, and children’s activity levels are also known to decrease as they age [9–11]. Establishing higher levels of physical activity in preschoolers may therefore be important.

Although parents provide the majority of preschool-aged children’s care, children under 5 years now spend increasingly large amounts of time in out-of-home care [12–15]. In consequence, home and childcare environments may both exert a large influence on young children’s health behaviors (including physical activity [16, 17]). International evidence suggests that low levels of MVPA [18] and high levels of sedentary behaviour [19] are common in preschool-aged children when in care, with much of this research conducted in the USA, Europe and Australia [18–20]. Uptake of childcare differs between these countries, with approximately 90 % of children attending in mainland Europe [13] in contrast to 49 % of 3–5 years olds in Australia [14] and 61 % of preschoolers aged 3–4-years-old in the USA [15]. Childcare attendance may also be influenced by maternal employment, as evidenced by trends in the USA which suggests that 81 % of US children with mothers in employment are cared for in childcare centers compared with only 17 % with unemployed mothers [15].

In the UK, 3–4-year-olds are entitled to receive 15 h per week of free childcare [21], and around 96 % of all eligible 3–4-year-olds benefitted from funded early years education in 2013 [12]. Most children therefore attend childcare, regardless of their parents’ formal employment status [22]. In addition, although UK childcare uptake is similar to that seen in Europe, differing types of provision and variation in childcare policies make comparisons across countries difficult [16]. However, notwithstanding these differences, existing studies to date have only considered children’s overall activity levels [6, 7], activity when children were in care [19, 23–25], or the influence of time spent in care [26, 27]. Where preschool-aged children accumulate their activity throughout the day, and how their activity levels differ by location is still therefore largely unknown.

Using objectively measured physical activity data matched to children’s location, we investigate the in-care and out-of-care activity patterns in a sample of 3–4 year-old British preschool-aged children, and explore differences in children’s activity level by childcare attendance.

## Methods

### Study design

The “Studying Physical Activity in preschool-aged Children and their Environment (SPACE) Study” is a cross-sectional childcare-based observational study. We recruited 275 3–4-year-old children and their parents through preschool and nursery settings in the Cambridgeshire area. Data were collected between January and July 2013. The University of Cambridge Psychology Ethics Committee provided ethical approval for the study (Pre.2012.68).

### Recruitment

We obtained a list of government-funded preschools and private nurseries (hereafter ‘settings’ to reflect the different types of childcare included in the study) in Cambridge from a government website (Ofsted; January 2013). We stratified settings by type (preschool or nursery) and by tertile of index of multiple deprivation (IMD; an area-level measure of deprivation [28]). Within these six strata childcare settings were invited to participate in writing and approached at random (using random digit allocation) (*n* = 88). A £50 voucher and feedback about their nutrition and activity environment were offered as incentives. Setting managers provided full written consent for participation (*N* = 30).

**Fig. 1:**
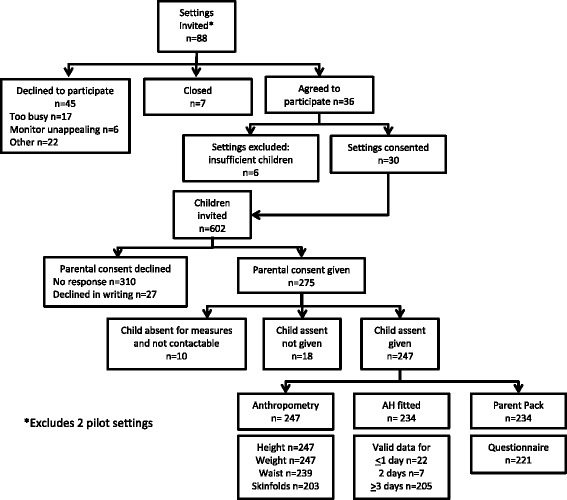
Flow chart of SPACE participant recruitment and participation rates

We sent an information pack to parents of preschool-aged children attending participating settings. Children were eligible to participate (*n* = 602) if they: were aged 3 or 4 years; were free from physical disability; attended the setting for at least 9 h per week (to ensure children spent >50 % of their government-paid allocation at that particular setting); and were registered to attend the childcare setting on the designated measurement day. A minimum of ≥5 children per setting with valid written consent (by a parent/legal guardian) was required to ensure sufficient analytical power. Children provided verbal assent prior to measurement (see Fig. 1).

### Data collection

Measurements were conducted at childcare settings, though home visits were offered if a child was absent on the measurement day. To assess children’s free-living activity, we fitted children with an Actiheart activity monitor (Cambridge Neurotechnology Ltd, UK), a combined lightweight heart-rate monitor and accelerometer, previously validated in preschool-aged children [29, 30]. The unit was secured to the chest using electrode pads, and set to record at 15 s epochs [31, 32]. We encouraged children to wear the monitor continuously for up to 7 days, including during water-based activity and sleep, and parents received written instructions regarding monitor use. Parents completed a questionnaire based on a previously validated measure [33]; questionnaires and monitors were collected from the setting 1 week later.

### Outcome variables

We used only accelerometry data here, as combined heart-rate data has been shown to explain little additional variation in estimates of free-living physical activity in preschoolers [29]. Counts data from the Actiheart monitors were downloaded and processed using STATA 13/SE [34]. To reflect when children were most likely to be active and/or in care, we used time-stamped accelerometry data between 7 am and 6 pm (maximum 660 min). Although children would plausibly be awake outside these hours, they were not, according to parental report, in care. We removed data periods of 100 min or more with zero-activity counts [35, 36], and days with <600 min of recording [37]. Average wear time was (mean ± SD) 6.7 ± 1.4 days, for 649.5 ± 46.6 min/day. We converted Actiheart counts to those of the Actigraph 7164 accelerometer (Actigraph, Pensacola, FL, USA) by using a conversion factor of 5 [38]. Conversion between monitor types is required due to differing frequencies of piezoelectric element used to measure acceleration. The conversion factor used here is based on laboratory experiments where both Actigraph and Actiheart monitors were worn simultaneously during treadmill walking and running, and for free-living activities [38, 39], and has been used previously in children of the same age [8]. We used validated cut points [40] to classify children’s activity as sedentary (SED: <38 counts per 15 s); light (LPA: ≥38–420); and moderate-to-vigorous (MVPA: ≥421) [40].

We aggregated all epochs within a 15-min segment and subsequently summed them for each hour. Outcomes were therefore expressed as average minutes per hour (min/h) a child spent at each activity intensity.

### Childcare exposure variables

We obtained data about a child’s childcare attendance for each day during the measurement week from the parental questionnaire, using a specifically designed question (see Additional file 1). Parents were asked “In a usual week, when does your child attend childcare? *Please only include care for your child taking part in SPACE and include regular formal and informal care* (e.g. *grandparents*, *friends* etc.).” Parents were provided with example responses and included their responses as free text, which we then coded for analyses.

To enable 1:1 matching with accelerometry data, we processed location data in the same 15-min intervals. We categorized free text answers ‘nursery’, ‘preschool’ and ‘childminder’ as in formal care (hereafter ‘in care’). We considered categories referring to parents (i.e. ‘mummy’, ‘daddy’, ‘us’ etc.), ‘at home’, ‘with grandparents’ or ‘Nanny’ as at home/informal care (hereafter ‘at home’), along with all time periods when parents did not specify that their child was in care. To undertake within-child comparisons, for each day we then derived three exposure variables: 1) the number of hours spent in care (continuous); 2) childcare attendance on the day (categorical: full-time (>5 h), part-time (1–5 h) or not in care (0 h)); and 3) in care vs. at home location during each of the 15-min blocks (dichotomous).

### Additional variables

We obtained hour, time of the day and week (weekday/weekend) and season (winter: December–February; spring: March–May; summer: June–August) from the Actiheart output. As preschool-aged children’s activity differs over the course of the day [8, 41], we selected time periods to explore the association between location (in care/at home) and activity throughout the day (see below).

Trained researchers recorded each child’s sex, measured height to the nearest 0.1 cm using a Leicester stadiometer, and weight to the nearest 0.1 kg using Seca digital scales in light indoor clothes, which we used to calculate children’s BMI and BMI z-score [42]. We used BMI z-score and child’s age in months at measurement (calculated from parental reported date of child’s birth) to describe child’s weight as thin, normal or overweight/obese using the International Obesity Task Force classifications [43]. Data on child’s ethnicity, mode of travel to childcare, and mothers’ and fathers’ self-reported age, BMI, educational and employment status were taken from the parental questionnaire.

### Statistical analysis

Analyses were conducted using STATA 13/SE [44]. A significance level of 0.05, set a priori, was used for all tests, and we used a two-tailed test to explore the direction of the relationship (i.e. whether the mean was either greater or less than x and therefore that the test statistic was in the top or bottom 2.5 % of its probability distribution). Independent *t*-tests were used to compare descriptive statistics for children with and without valid activity data (where valid data were defined as ≥2 days with 600 min or more of physical activity data), and children’s average activity levels by sex and time of the week.

We conducted a series of two- and three-level hierarchical linear regression analyses to explore the within-child differences in physical activity by childcare attendance. Analyses were adjusted for children’s age in months, maternal education status, mode of transport to childcare and season. Hierarchical regression allows for within-child clustering of activity behaviour and between-child variation in activity levels [45]. As children’s activity did not cluster within setting (ICC_LPA_:0.003; ICC_MVPA_:0.05) it was not entered as a cluster variable in hierarchical analyses.

We conducted two-level hierarchical regression analyses to assess the influence of daily time spent in care on within-child activity levels, expressed as min/h spent SED, in LPA and MVPA (Level 1: daily activity; Level 2: child). First, we used the continuous exposure variable to assess if there was a linear association between daily hours in care and activity. We then used the categorical variable to determine how activity differed on days children were not in care, were in care part-time (1–5 h) or full-time (>5 h). Finally, we conducted a series of three-level regression analyses to explore whether children’s in-care activity levels were the same as activity levels when at home at the same time of the day (e.g. to determine if children were more active when in care or at home between the hours of 9 am–12 pm). We assessed five time periods: daily (7 am–6 pm); early mornings (7–9 am); mornings (9 am–12 pm); afternoons (2–3 pm) and early evenings (3–6 pm). Morning and afternoon time periods were derived as formal ‘preschool’ sessions in the UK are most likely to take place during this time (Level 1: min/h of activity in and out of care; Level 2: day; Level 3: child).

We stratified all regression analyses by sex, due to a significant sex by care interaction (*p* = 0.004), indicating that there was a greater difference in activity levels by location (home vs. care) for boys compared to girls. There were no significant interactions between sex and type of care, time of day or maternal employment. Although there was a significant difference in children’s overall activity on weekdays vs. weekend days (7 am–6 pm: 512.2 vs. 499.1 min; paired *t*-test = 2.3, *p* = 0.02), children’s average activity levels on weekday non-care days did not differ from those on weekend days (492.4 vs. 499.1 min, *t* = −1.2, *p* = 0.22). Weekend days were therefore classified as non-care days and included in analyses.

Sensitivity analyses assessed: a) how removing data relating to locations classified as ‘childminder’ influenced our findings as these setting may be considered similar to those classified as ‘at-home’; and b) the influence of limiting our analysis sample to children with at least 1 weekend and 1 weekday (*n* = 197).

## Results

### Sample descriptives

In total, 275 children had valid parental consent, of whom 234 (85 %) were fitted with the Actiheart monitor and 221 (80 %) returned the questionnaire (Fig. 1). Of these, 202 children (51 % female) had valid physical activity data (for ≥2 days), and were included in analyses (Table 1). Compared to children who provided valid accelerometry data, those who did not (*n* = 22) were slightly younger (45.6 (SD: 6.0) vs. 47.5 (5.0) months; *p* = 0.02), but did not differ significantly by sex, weight status, maternal education or parental employment.

**Table 1:** Descriptive characteristics of children included in analyses (*n* = 202)

	Boys	Girls
Child Characteristics		
*N* (%)	99 (49)	103 (51)
Age (in months)	47.4 (5.2)	47.7 (4.8)
Ethnicity (*N* (%))		
White British	70 (70.7)	80 (77.7)
White Other	9 (9.1)	11 (10.7)
Mixed Ethnicity	13 (13.1)	6 (5.8)
Other	7 (7.1)	6 (5.8)
BMI z-score	0.47 (0.97)	0.30 (1.03)
Weight category^a^ (*N* (%))		
Normal	83 (83.8)	87 (84.5)
Overweight	13 (13.1)	11 (10.7)
Obese	3 (3.1)	5 (4.9)
Average daily hours in childcare	5.8 (2.5)	5.4 (2.5)
Maternal Characteristics		
Age (in years)	37.7 (5.0)	37.2 (5.6)
BMI (in kg/m^2^)	24.0 (4.3)	24.2 (4.7)
Education (*N* (%))		
GCSE/A-levels	26 (28.3)	35 (35.7)
Degree	28 (30.4)	34 (34.7)
Higher degree	42 (45.7)	29 (29.6)
Hours worked per week^b^ (*N* (%))		
Not employed	26 (28.6)	26 (26.0)
< 20 h	17 (18.7)	15 (15.0)
21–35 h	28 (30.1)	31 (31.0)
> 35 h	20 (22.0)	28 (28.0)
Paternal Characteristics		
Age (in years)	39.5 (5.5)	39.9 (8.2)
BMI (in kg/m^2^)	25.3 (3.5)	25.2 (3.3)
Paternal Education (*N* (%))		
GCSE/A-levels	22 (25.6)	26 (29.9)
Degree	22 (25.6)	26 (29.9)
Higher degree	42 (48.8)	35 (40.2)
Hours worked per week^b^ (*N* (%))		
< 40 h	25 (29.8)	30 (35.3)
≥ 40 h	59 (71.2)	55 (64.7)

All values mean (SD) unless stated otherwise; *BMI* Body mass index, *GCSE* General Certificate of Secondary Education, *A*-*levels*, Advanced Levels

^a^Weight category derived using the International Task Force on Obesity cut points

^b^categorized based on distribution

### Children’s activity and associations with childcare attendance

Between 7 am and 6 pm, children engaged in 506.6 (SD: 104.0) min of activity on average, with boys being significantly more active than girls (LMVPA: 518.3 (103.0) vs. 495.1 (104.3) min, *t* = 2.3, *p* = 0.02; MVPA: 233.2 (129.5) vs. 197.9 (121.8) min, *t* = 2.9, *p* = 0.004). All children met current activity guidelines on all measurement days. Children spent between 2 and 11 h in care on any given day.

Table 2 shows results of the two-level regression analyses exploring the association with time in care. Each additional hour in care was associated with 0.5 (SE: 0.1) and 0.6 (0.1) fewer min/h spent SED in boys and girls respectively. There was a positive association of a similar magnitude with boys’ hourly minutes of MVPA and girls’ LPA. Comparing activity levels on days at home with days in care part-time or full-time, children were significantly less SED and engaged in more MVPA on days when in care (either part-time or full-time). There was no association with LPA.

**Table 2:** Association between time spent in care and children’s physical activity

	Activity intensity β (95 % CI)		
	Sedentary	LPA	MVPA
Hours in care^^^			
Boys	−0.5 (−0.7, −0.3)^***^	0.1 (−0.1, 0.3)	0.4 (0.1, 0.7)^**^
Girls	−0.6 (−0.8, −0.4)^***^	0.5 (0.3, 0.6)^***^	0.1 (−0.1, 0.4)
Time in care			
Boys			
Part-time (vs. no care)	−1.6 (−3.1, −0.1)^*^	−0.3 (−1.5, 0.8)	1.8 (0.0, 3.7) ^*^
Full-time (vs. no care)	−4.9 (−6.3, −3.4)^***^	−0.1 (−1.2, 1.0)	5.0 (3.2, 6.9)^***^
Full-time (vs: part-time)	−3.3 (−5.0, −1.5)^***^	0.4 (−1.2, 1.5)	3.2 (1.0, 5.4)^***^
Girls			
Part-time (vs. no care)	−1.6 (−3.2, −0.2)^*^	−0.2 (−1.3, 0.9)	1.8 (0.1, 3.4)^*^
Full-time (vs. no care)	−3.0 (−4.5, −1.5)^***^	0.0 (−1.0, 1.1)	3.0 (1.4, 4.5)^***^
Full-time (vs: part-time)	−1.4 (−3.2, 0.5)	0.3 (−1.0, 1.5)	1.2 (−0.8, 3.1)

Analyses use 687 observations for girls, 705 observations for boys; β: minutes per hour spent sedentary or active on days in care vs. reference category; 95 % CI: 95 % confidence interval; *p < 0.05; **p < 0.01; ***p < 0.005

^^^β: minutes per hour spent sedentary or active for each one hour increase spent in care

**Fig. 2:**
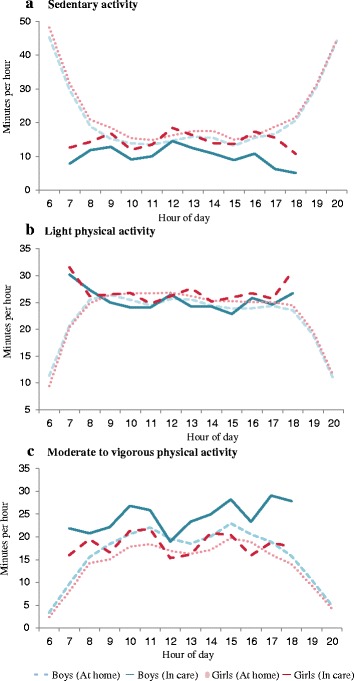
Children’s daily average hourly physical activity levels by intensity and location. **a** Sedentary time. **b** Light physical activity. **c** Moderate to vigorous physical activity

Lastly, we assessed how activity levels at the same time of the day differed between in care and at home (Table 3; Fig. 2). Over the whole day both boys and girls were less SED, and engaged in more LPA and MVPA when in care compared to at home. This difference was most pronounced in the mornings (7–9 am), with associations somewhat attenuated when analyses were restricted to 9 am–12 pm. Few differences between activity levels in and out of care were seen after 12 pm, except for boys’ activity levels between 3 and 6 pm, where boys engaged in 5.4 (0.9) min less sedentary and 4.7 (0.8) min more MVPA.

**Table 3:** Influence of being in care vs. at home on children’s physical activity

	Activity Intensity β (95 % CI)		
	Sedentary	LPA	MVPA
Boys			
7 am–6 pm^a^	−6.4 (−7.4, −5.5)^***^	0.6 (−0.1, 1.3)	5.7 (4.8, 6.7)^***^
7–9 am^b^	−13.8 (−16.5, −11.2)^***^	3.6 (1.7, 5.6)^***^	10.1 (8.0, 12.2)^***^
9 am–12 pm^c^	−3.3 (−4.8, −1.7)^***^	−1.0 (−2.3, 0.4)	4.2 (2.2, 6.2)^***^
12–3 pm^d^	−1.4 (−3.0, 0.2)	−0.2 (−1.4, 1.0)	1.4 (−0.3, 3.1)
3–6 pm^e^	−5.4 (−7.0, −3.7)^***^	0.3 (−1.0, 1.6)	4.8 (3.0, 6.7)^***^
Girls			
7 am–6 pm^a^	−4.8 (−5.8, −3.8)^***^	1.8 (1.1, 2.5)^***^	3.0 (2.1, 3.8)^***^
7–9 am^b^	−10.7 (−12.9, −8.4)^***^	3.2 (1.2, 5.2)^**^	7.9 (5.7, 10.0)^***^
9 am–12 pm^c^	−3.1 (−4.7, −1.4)^***^	−0.2 (−1.5, 1.1)	3.5 (1.8, 5.2)^***^
12–3 pm^d^	−1.3 (−2.8, 0.5)	0.3 (−0.9, 1.4)	1.0 (−0.6, 2.5)
3–6 pm^e^	−1.2 (−2.9, 0.5)	1.2 (−0.1, 2.5)	−0.2 (−1.8, 1.5)

^a^ *n* = 1088 observations for boys, *n* = 1051 for girls; ^b^ *n* = 704 for boys, *n* = 682 for girls; ^c^ *n* = 716 for boys, *n* = 699 for girls; ^d^ *n* = 783 for boys, *n* = 745 for girls; ^e^ *n* = 752 for boys, *n* = 763 for girls; β: minutes per hour spent sedentary or active when in care compared to at home during hours specified; 95 % CI: 95 % confidence interval; *p < 0.05; ** *p* < 0.01; *** *p* < 0.005

Removal of data with locations classified as ‘childminder’ and limiting analyses to children with at least 1 week and weekend day did not alter our findings.

## Discussion

We showed that 3–4 years-old children were less sedentary and more active on average when in care compared to at home. This effect was larger in boys than in girls, in the (early) mornings for both, and afternoon for boys. Comparison of full- vs. part-time days indicated that this effect appeared to be cumulative over the day. Although all children comfortably met current physical activity guidelines, sedentary activity when at home appeared to be replaced by higher intensity activity when in care. This work provides important novel information about the differential impact of place and time on preschool-aged children’s activity, and adds to the current limited research assessing the contribution that childcare attendance plays.

We observed high activity levels overall in this sample, with all children meeting current activity guidelines of 180 min of LMVPA. This and previous work in UK preschoolers [8] therefore suggests a more positive picture of preschool-aged children’s activity levels than reported elsewhere [19]. These differences are in part likely due to heterogeneity between samples, differences in monitor type, wear positions and data processing. A large proportion of children’s time here was however spent in light intensity activities, for which the health benefits are currently unknown. Children engaged in less MVPA, despite higher intensity activity being associated with more favorable outcomes, including lower fat mass [35], in preschoolers [35, 46]. Children’s MVPA in childcare also appeared to be replaced with sedentary time out of care, with the latter independently associated with poorer health outcomes in this age group [47]. Therefore, despite children ‘meeting activity guidelines’, implementing policies that encourage higher intensity activity in young children, regardless of their location, may prove to be clinically important for young children’s health. This is particularly true given almost a quarter of UK and US preschool-aged children will be classified as overweight or obese by their 5th birthday [48, 49].

In contrast to previous studies [26, 27], hours in care were inversely associated with children’s sedentary time and positively associated with MVPA. We also found differences in MVPA of up to 5 min per hour between in-care and at-home activity, representing a potentially large difference in physical activity over the course of a day. Morning childcare attendance appeared to be particularly conducive to children’s activity, with no compensation (i.e. higher levels of at-home activity) later in the day. Such differences may occur because settings provide greater active opportunities, or because parents perceive that childcare is responsible for providing adequate activity [50]. As noted, decreased MVPA at home appeared to be replaced by sedentary time suggesting that parents may let their children rest or engage in more sedentary activities when at home [51]. Moreover, though increasing evidence suggests that parents’ [52–54] (and siblings’ [55]) activity levels are associated with those of their preschool-aged children, parents suggest they have little time to participate with their child [56], and levels of physical activity are also known to be lower in parents of young children [57]. Promotion of home-based activity to limit sedentary time and encourage higher-intensity activity within families may therefore be advantageous. With appropriate training, this may be facilitated by childcare settings, given their reach to young children and families [12]. Indeed, both settings and parents in the SPACE study stated they would welcome information about how to better encourage activity within families, and with their children respectively, suggesting there is a current unmet need in this area.

As time spent in childcare appeared to be conducive to higher intensity activity in this sample, this may suggest that UK ‘free-flow’ policies represent an important public health strategy. This policy allows children to choose their activities, freely moving between inside and outside environments for most of the day, regardless of weather conditions. Such policies, which may be piloted relatively easily on an initial basis, may be beneficial and could be considered in other countries where the childcare day is more structured and physical activity levels in preschoolers are lower [19].

Interestingly, there appeared to be larger differences between in-care and at-home activity for boys compared to girls: boys’ at-home activity was comparable to girls’, but boys’ in-care activity was far higher. Free flow policies may suit boys’ (innate [58]) activity preferences, with rough-and-tumble play [59] and use of wheeled toys [60] better facilitated in childcare. Girls’ preferences for light intensity activities, such as social play with peers [61] or dolls, or with art materials [60], likely vary less between the home and childcare environments. As child-led activities are a key component of UK early education, dissuading children from their self-selected activities is actively discouraged, which may reinforce these gender differences. Childcare providers’ and parents’ own beliefs and behaviours may also inadvertently influence children’s and girls’ activity [62, 63], with gender stereotyping of play shown to perpetuate sex differences far beyond early childhood [64, 65]. Awareness of these differences and greater encouragement of girls’ higher intensity activity may therefore be warranted, even from an early age.

### Strengths and limitations

Using an objective measure of physical activity, we provide novel information about the influence of childcare attendance on UK children’s activity. Use of time-stamped data allowed a more precise assessment of physical activity outcomes and within-child associations between location and activity levels. Multi-level regression facilitated analysis of a large number of observations per child, increasing our power to detect (small) significant, and meaningful, differences in activity, despite high overall levels. Completion of the parental questionnaire was high (94 %) allowing satisfactory matching of activity and location data; 14 % of parents indicated deviations from their child’s normal childcare routine, which were taken into account. A degree of misclassification of children’s locations during the measurement week cannot however be ruled out. We also acknowledge that our definition of ‘at home’ is likely to include other locations children visit with their parents/carers, such as supermarkets or playgrounds. As free-flow policies in the UK operate inside and out regardless of weather conditions (with children wearing waterproof clothing etc.), it is plausible that weather may have a greater effect on at-home activity. We partially accounted for this by adjusting for season, although weather-related residual confounding cannot be discounted.

Children were drawn from settings recruited from the top tertile of IMD scores in England [28], but no differences in IMD scores of settings that did and did not participate were found, suggesting included children were representative of the wider eligible population. The impact of including children from higher socio-economic backgrounds on findings is unknown. Yet socio-economic circumstances do not appear to be associated with preschoolers’ activity levels [66], and take-up of free preschool entitlement is not related to parental employment in the UK [22]. As preschool policies are similar across the UK, these results conceivably provide an indicative estimate of potential differences in a more generalizable population. That said, this was a childcare-based sample of predominantly White children, who were less likely to be overweight/obese compared to the national average [48], and care should be taken not to extrapolate these results to minority populations.

## Conclusions

This work adds to the limited evidence of how place and time influence preschoolers’ physical activity: we found that children engaged in higher levels of MVPA when in care compared to at home, and that this difference was larger for boys. Future research should identify factors accounting for this difference. UK childcare policies may also be conducive to young children’s MVPA, and could be considered as intervention strategies in other countries. Interventions among UK preschool-aged children may however consider targeting predominantly non-care time with the aim of reducing sedentary time and increasing children’s higher intensity physical activity.

## Additional file

Additional file 1:
**Specially designed question to assess childcare attendance.** (DOCX 16 kb)

## Abbreviations

UK: United Kingdom
SED: Sedentary time
LPA: Light Physical Activity
MVPA: Moderate-to-vigorous activity
SE: Standard error
USA: United States of America
SPACE: Studying Physical Activity in preschool Children and their Environment
IMD: Index of Multiple Deprivation
BMI: Body Mass Index
ICC: Intra-class Correlation Co-efficient
LMVPA: Total Physical Activity
