# Supplementary material for: UK Preschool-aged children’s physical activity levels in childcare and at home: a cross-sectional exploration
Source: Int J Behav Nutr Phys Act. 2015 Sep 26;12:123. doi: 10.1186/s12966-015-0286-1 (PMC4583748; doi:10.1186/s12966-015-0286-1)
Supplement: Additional file 1: — Specially designed question to assess childcare attendance. (DOCX 16 kb) [file 12966_2015_286_MOESM1_ESM.docx]

### Supplement: Example of specially designed question to assess childcare attendance

In a usual week when does your child attend childcare?

*Please only include care for your child taking part in SPACE and include regular formal and/or informal care (grandparents, friends etc.)*

|  | **Time** | **Type of Care** |
| --- | --- | --- |
| *Example* | 9-12 noon | Preschool |
| *Example* | 12-2 pm | Child-minder |
|  |  |  |
| Monday |  |  |
|  |  |  |
|  |  |  |
| … |  |  |
|  |  |  |
|  |  |  |
| Weekend |  |  |
|  |  |  |
|  |  |  |
